# Identification of novel targets for breast cancer by exploring gene switches on a genome scale

**DOI:** 10.1186/1471-2164-12-547

**Published:** 2011-11-03

**Authors:** Ming Wu, Li Liu, Christina Chan

**Affiliations:** 1 Department of Computer Science and Engineering, Michigan State University, East Lansing, MI 48824, USA; 2 Department of Microbiology & Molecular Genetics, Michigan State University, East Lansing, MI 48824, USA; 3 Department of Chemical Engineering and Material Science, Michigan State University, East Lansing, MI 48824, USA; 4 Department of Biochemistry and Molecular Biology, Michigan State University, East Lansing, MI 48824,USA

## Abstract

**Background:**

An important feature that emerges from analyzing gene regulatory networks is the "switch-like behavior" or "bistability", a dynamic feature of a particular gene to preferentially toggle between two steady-states. The state of gene switches plays pivotal roles in cell fate decision, but identifying switches has been difficult. Therefore a challenge confronting the field is to be able to systematically identify gene switches.

**Results:**

We propose a top-down mining approach to exploring gene switches on a genome-scale level. Theoretical analysis, proof-of-concept examples, and experimental studies demonstrate the ability of our mining approach to identify bistable genes by sampling across a variety of different conditions. Applying the approach to human breast cancer data identified genes that show bimodality within the cancer samples, such as estrogen receptor (ER) and ERBB2, as well as genes that show bimodality between cancer and non-cancer samples, where tumor-associated calcium signal transducer 2 (TACSTD2) is uncovered. We further suggest a likely transcription factor that regulates TACSTD2.

**Conclusions:**

Our mining approach demonstrates that one can capitalize on genome-wide expression profiling to capture dynamic properties of a complex network. To the best of our knowledge, this is the first attempt in applying mining approaches to explore gene switches on a genome-scale, and the identification of TACSTD2 demonstrates that single cell-level bistability can be predicted from microarray data. Experimental confirmation of the computational results suggest TACSTD2 could be a potential biomarker and attractive candidate for drug therapy against both ER+ and ER- subtypes of breast cancer, including the triple negative subtype.

## Background

**Figure 1:**
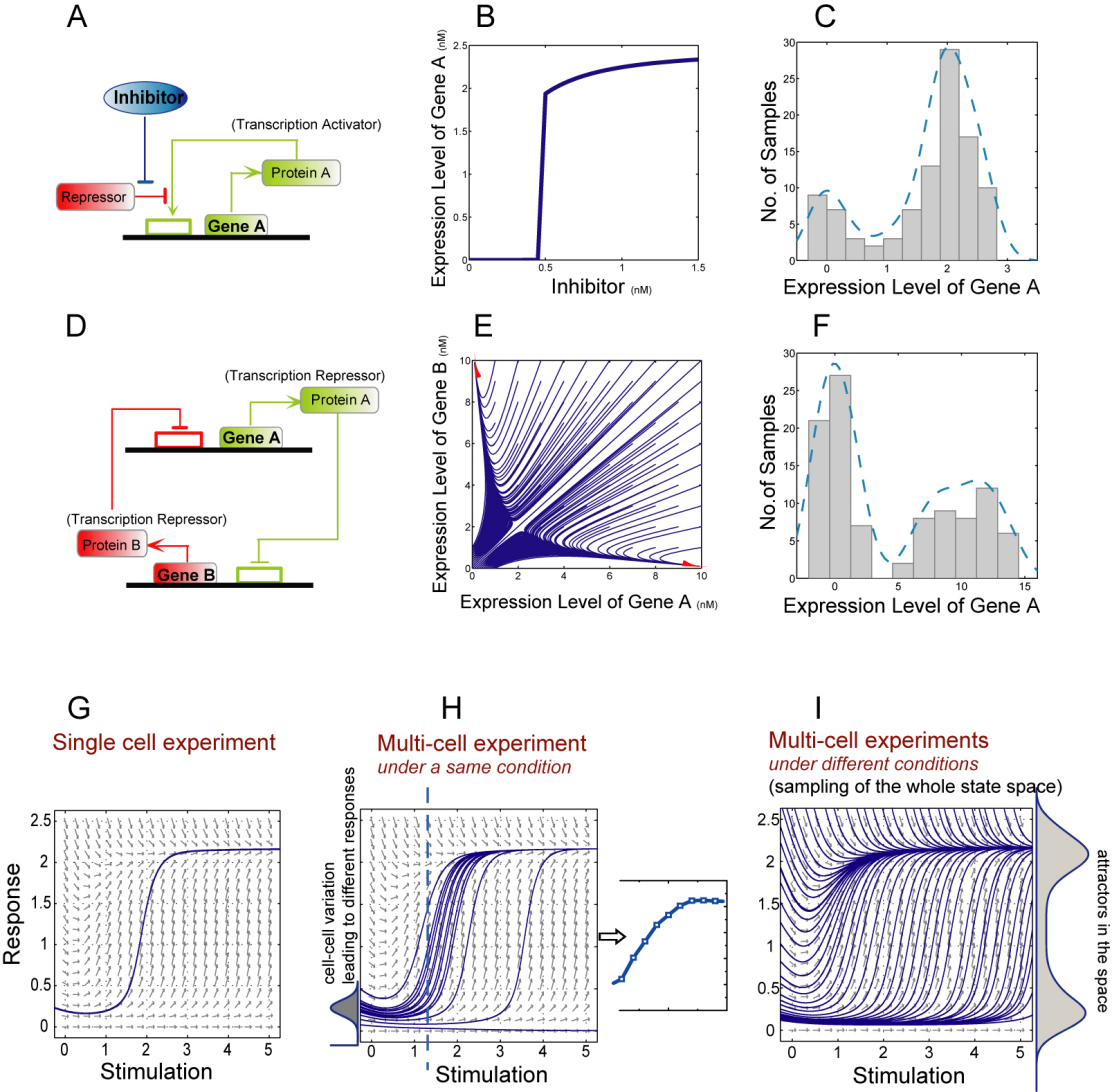
**Dynamics of gene switches and bimodality in their expression profiles**. **A) **A synthetic genetic switch [24] that contains a repressed positive feedback is stimulated by an inhibitor of the repressor. **B) **The stimulation-response curve of the genetic circuit. **C) **The histogram of the steady-state gene expression level of 100 random sample simulations of the genetic switch shown in A. Simulations are performed with randomly (uniformly) generated initial conditions (initial gene expression level) and 20% Gaussian variance in the parameters. **D) **A synthetic genetic toggle switch [16] that contains double negative feedbacks. **E) **The state space of the gene expression levels. Each trajectory (blue lines) is the response curve with respect to a particular initial condition. The red arrows represent the asymptotes of the response curves, i.e. all trajectories converge to the two attractor-states. **F) **The histogram of the steady state gene expression level of 100 random sample simulations of the genetic switch shown in B. Simulations are performed with uniformly generated initial conditions (initial gene expression level) and 20% Gaussian variance in parameters. **G) **Schematic representation of a phase plane of a gene switch. Single cell dose response experiments should be able to measure the response curve and uncover the switch-like behavior. **H) **Experimental measurements of the average expression level of a cell population will mask the switching behavior. A Gaussian distribution is plotted to represent the cell-cell variances in the population. Different cells, according to their initial gene expression level, could have different response curve (blue trajectories). The averaging of the variation in the responses results in a seemingly graded response. **I) **Experiments across a range of different conditions allowing for the sampling of a large state space recover the switch-like behavior. Each sample could fall in the neighborhood of a possible steady state (points on the blue trajectory). The steady states (on/off states of the gene switch) are the dense regions of the possible response curves in the state space, i.e. the samples occurs at higher frequencies in these states, which results in a bimodal distribution in the observed profiles.

Given the complexity of gene regulatory networks, knowledge of the properties of individual components in the network are not sufficient to elucidate the cell physiology. Thus systems biology has evolved to uncover "emergent properties" that arise from the intricate interactions of gene networks. One such emergent property, "switch-like behavior" or "bistability", describes a dynamic feature of a particular gene [1] to preferentially toggle between two steady-states. Multiple steady states are often observed in chemical and biochemical reactions (reviewed by [2]) and are characterized by a non-linear response. Bistability happens to be a special case involving two steady-states, giving rise to a "switch-like behavior". In biochemical reactions, such "bistable" behavior shows a sharp sigmoid function or a hysteresis structure (see examples in Figures 1B and 1E), whereby the state of the variable flips between high and low levels. Such an "all-or-none" state transition usually depends on a threshold, i.e., the concentration of the stimulator or regulator. Hysteresis depends further on the previous state of the system.

The expression level of a gene switch does not change gradually but rather has two distinct steady-states: HIGH or LOW, ON or OFF, ALL or NONE. The ability of switches to convert a graded signal into a binary response ensures that a cell responds in a decisive manner or unambiguously commit to a specific program [3]. Furthermore switches have been noted for their noise-filtering capacity. Endogenous noise are typically lower for fully repressed or induced expression states than in a gene where the state changes continuously [4,5].

Bistable behavior of gene switches have been reported to play pivotal roles in many important aspects of cell physiology, including cell fate decisions, cell cycle control, and cellular responses to environmental stimulation [6,2]. E. coli *lac *operon is a famous gene switch that uses a hysteretic feedback to decide between glucose and lactose utilization [7]. Many bistable systems have been discovered in bacteria, including the genetic transformation in *Bacilius subtillis *and sporulation in many bacterial species [8]. In mammalian systems, gene switches and bistability have been postulated as the underlying mechanism for cellular differentiation, but rarely has this been confirmed experimentally, until recently with the work on neutrophil differentiation [9]. Another interesting observation is that cells have "memory", and hysteresis has been shown to govern short-term memory in lymphoid cells, preserving information of past encounters with antigen [10]. Thus, the discovery of gene switches in cellular responses has become a milestone in molecular biology and prompt strong interest in understanding the function and design of gene networks [7].

Despite the importance of gene switches, identifying multiple steady-states, and in particular switches, has been difficult. Our understanding of gene switches has been mostly based on simulations of generic feedback circuits and well-characterized biological modules [11-14]. Theoretical studies of feedback circuits have elucidated general principles of network dynamics, but they usually lack solid evidence to associate these principles with real physiological processes in cells. Few studies have succeeded in demonstrating functional roles of actual switches in biological systems by coupling detailed kinetic modeling with rigorous experimentation [15,10]. This is because well-characterized models with equations and kinetic parameters are difficult to obtain for real, complex biological systems, in part because current techniques are not able to quantitatively measure reaction constants at the single-cell resolution for all the network components. Alternatively, researchers in synthetic biology have designed artificial gene networks with specific functions and implemented the interactions by manipulating or bringing together exogenous genetic components [16-18]. Thus current methods of experimentally studying switches have been limited to well-characterized or synthetic small modules.

Switches play a central role in cell decision, and the ability to predict whether switches can occur without a *priori *detail information of the network would be significant. For instance, the ability to identify which genes are turned on or off in cancer versus normal cells would have a tremendous impact on identifying the most pertinent molecular signatures or targets for drug therapy. Therefore a major challenge confronting the field, which we address in this study, is how to effectively identify gene switches or bistable states by mining high-throughput data.

Previous approaches addressed this question by analyzing the network topology. These studies assume that bistability requires particular feedback structures [19,3], and discovered dynamic features by searching for these structures (e.g. positive feedbacks) in protein-protein interaction and protein-DNA interaction networks [20]. However, these feedback structures do not ensure switch-like behavior. From modeling and simulations of genetic circuits, positive feedback (or even feedback itself) has been shown to be neither necessary [21,6] nor sufficient [22] to ensure switch-like behavior. Furthermore, it is less likely that one can uncover a dynamic property from static networks.

Alternatively, we theorize that the dynamic "behavior" of a switch could be identified by analyzing the gene expression profiles from a wide range of conditions. We propose a top-down mining approach to identify gene switches from microarray gene expression data. Taking advantage of the tremendous amount of expression data, our approach aims to identify bimodality, which we hypothesize is an essential characteristic of a gene switch. We perform theoretical analysis and provide proof-of-concept applications on both synthetic and yeast microarray datasets. We further apply our methodology on an integrated human expression dataset to probe the characteristic signatures of human cancer and confirm that our approach is able to identify a gene with switch-like behavior. To the best of our knowledge, this is the first attempt at applying mining approaches to explore gene switches on a genome-scale.

Since the state of gene switches in the genetic network governs the phenotype [23], we postulate that recognizing specific gene switches will enable one to identify biomarkers or molecular signatures that would be better drug targets for treating a disease. We demonstrate the utility of our mining approach in human breast cancer by analyzing a paired breast cancer/normal tissue expression dataset against the integrated human gene expression dataset. We uncover two types of potential gene switches in breast cancer, with one type (denoted as Type 1) showing bimodality within the breast cancer and a second type (denoted as Type 2) showing predominantly one modality in breast cancer.

Known therapeutic targets for breast cancer are uncovered under the Type 1 genes, such as estrogen receptor (ER, or ESR) and human epidermal growth factor receptor 2 (ERBB2, or HER2/neu), which are identified as gene switches for this cancer, and their bimodality in the cancer samples represent well-known subtypes in breast cancer. The other type of gene switch shows predominantly one modality in the breast cancer samples, and is where we discover the TACSTD2 (Trop2) gene. The expression of TACSTD2 is turned OFF in most normal samples but ON in almost all of the breast cancer samples independent of the subtypes. We predict through sequence matching of transcription factor (TF) sites that CREB could regulate TACSTD2, thereby implicating a novel transcriptional mechanism by which TACSTD2 is regulated. Our experimental studies on multiple breast cancer cell lines confirm the switch-like behavior of TACSTD2 and provide evidences for the transcriptional regulation of the gene. These results demonstrate the ability of our mining approach to identify gene switches that could be candidate biomarkers and novel therapeutic targets in breast cancer.

## Results

A gene switch has two steady states, which will produce a bimodal distribution in its expression profile when sampled across different conditions. Figures 1-A and 1D show the gene network topology of two typical regulatory circuits that exhibit bistable behavior. Figure 1-A is a positive self-feedback transcriptional system under the control of a transcriptional repressor. Figure 1-D is a double-negative feedback system, also known as a toggle switch, which produces mutually exclusive activation of two genes. Both circuits have been synthesized and implemented in cell systems [24,16] to confirm their switching behavior. Simulations based on the kinetic models of these systems [24,16] (see details of the equations in the METHOD) confirm the on/off and toggle-like switching behavior in their response curve (Figure 1-B) and state space (Figure 1-E). By simulating random samples from a wide range of conditions with different initial states, this unique feature of two distinct steady-states of gene switches results in a gene expression histogram profile containing two modes (Figures 1-C and 1-F). This bimodality is observed despite the noise (20% Gaussian noise) imposed on the parameters.

A challenge in experimentally identifying gene switches is their population effect. In single cell experiments, if obtainable, the response curves would represent individual cell measurements, and a gene that switches will exhibit a steep jump between the steady states (Figure 1-G). However many biological measurements (RT-PCR, Western Blotting), including microarray analysis, provide the population-average. In fact, even with single cell measurements, individual clones can contribute cell-cell variances, with differences in the protein expression levels across different cells. In Figure 1-H the cell-cell variance is modeled by a Gaussian distribution in the protein expression and different cells in a clone would then respond differently to stimulation, leading to a continuous change in the averaged response curve (see small graph in Figure 1-H). This explains, in part, the difficulty in identifying switches through standard experiments.

We proposed that an unbiased sampling across a range of different conditions could address this issue and help reveal the dynamic feature of gene switches. In Figure 1-I we show analytically, potential response-curves (the blue trajectories) in the whole state space of a gene switch. Each sample within the system would asymptotically approach one of the two possible steady states (dark blue region). Since the on/off states are the steady states which most cells will concentrated in upon stimulation, the samples will have higher probability of staying in these states, leading to a bimodal distribution in the observed expression profiles. We use a ΔAIC value (see METHOD) to capture whether a gene is likely to show multistable behavior. Compare with simpler criteria, such as separation and kurtosis [25], ΔAIC is more reliable and more resistance to noisy data (Additional file 1, Figure S1). The ΔAIC value is computed by comparing the goodness of fit of the data to either a Gaussian mixture model or a single Gaussian model, and assesses whether a bimodal distribution can explain the data better.

### A Proof-of-Concept application of the E2F-Rb network

The E2F-Rb network is a well-characterized system in mammalian cell fate determination, whereby the Retinoblastoma (Rb) protein regulates the transcriptional factor, E2F, to control the restriction point for the G1-S transition in cell cycle [26]. A simplified kinetic model was constructed for the E2F-Rb system [15], in which two genes Myc and CycD (Cyclin D: Cdk4,6) are activated by sufficient growth signal (serum) to induce E2F activation, which then directs the synthesis of downstream factors, such as CycE for DNA replication. The E2F self-activation and CycE-mediated E2F activation constitute two positive feedbacks in the system. It then was experimentally [15] confirmed that the level of E2F switches ON or OFF for cell-proliferation and cell-cycle arrest, respectively, suggesting E2F acts as a gene switch, while CycD and Myc do not show such switch-like behaviors.

**Figure 2:**
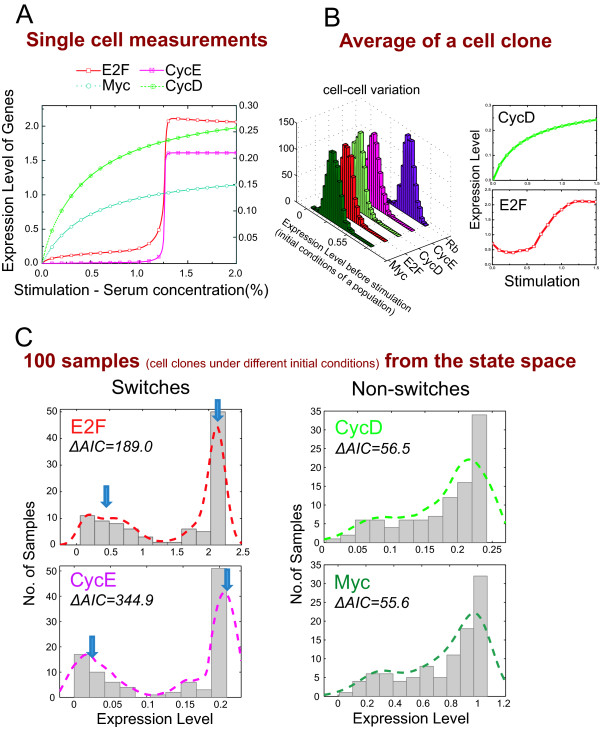
**Proof-of-concept example: simulation of the E2F-RB network**. The network structure and kinetic model are obtained from [15]. **A) **Simulation of the kinetic model based on a fixed initial condition (provided in [15]) represents measurement at the single cell-resolution of the system. The response curves of serum stimulation on the different genes in the model are plotted. **B) **Assign a Gaussian distribution with small variances on the initial gene expression level of the untreated cells to represent the cell-cell variation in a clone. Simulation-results are computed by averaging the responses of 100 cells in a clone. **C) **Sampling of 100 clones under different, randomly generated initial conditions. The simulation results are shown as histograms of the expression level of the different genes, together with their ΔAIC values.

We perform simulations based on the kinetic model [15] to generate a synthetic gene-expression dataset. The stimulation-response curve of a single-cell is shown in Figure 2-A, and confirms a graded response for Myc/CycD and bistable dynamics for E2F. The downstream factor CycE, controlled by E2F, also shows a switch-like response. Introducing a distribution in the expression level to represent cell-cell variation within a clone, and averaging multiple simulations (Figure 2-B) shows that population averaging for any one condition disguises the switch-like behavior and is indistinguishable from a graded responses, which is consistent with previous RT-PCR experiments [15].

We then simulate 100 cell clones, each clone with a random initial condition, and measure the steady state expression level of the network components for each clone. In this way we synthetically generate 100 "microarrays" for 100 different conditions. It is clear genes that have two steady states, i.e. E2F and CycE (effector of the gene switch), exhibit two distinct modes in their expression profiles (Figure 2-C). Each gene's ΔAIC value is calculated from the synthetic expression data and the switches exhibit higher ΔAIC values than the non-switches. Thus ΔAIC can be used to rank and help uncover genes that are bistable.

### A Proof-of-Concept application to Yeast microarray data

**Figure 3:**
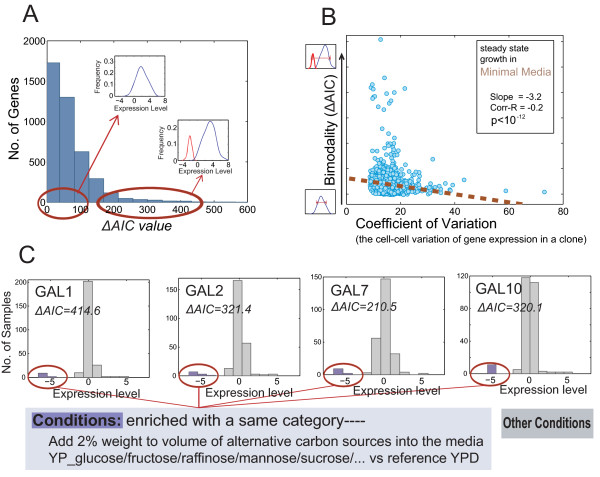
**A proof-of-concept application on the yeast dataset**. **A) **A histogram representing the distribution of ΔAIC value among the yeast genes. Most genes have small ΔAIC and exhibit an uni-modal expression profile. A relatively small number of genes have high ΔAIC and show bimodality in their expression. **B) **A negative correlation between bimodality (described by ΔAIC) and expression noise (as described by the coefficient of expression variation) in the yeast genes. **C) **The four GAL genes show bimodality and one of their modes are enriched within the same condition/category.

We apply our mining approach to an integrated yeast microarray dataset containing 500 yeast experiments (see METHOD) and calculate the ΔAIC value for each gene in the dataset. With such a large set of conditions, the ΔAIC value is fairly robust (see Additional file 1, Table S1, for a comparison of the ΔAIC ranking based on different sub-sets of the data). A histogram of the ΔAIC value among the yeast genes is shown in Figure 3-A. Most genes have low ΔAIC, and their expression appear unimodal. However, a few genes have high ΔAIC values and clearly show bimodality.

The genes with high ΔAIC values have distinct states under different conditions. By collating and comparing those conditions under the two distinct expression states, one can potentially identify the phenotypes in which the genes are functioning. Given that a phenotypic ontology is not available, it is difficult to compare conditions. Nevertheless, one approach is to categorize conditions by the type of perturbations, e.g. heat shock (with different temperature and length of time), hypo-osmotic shock (different time points), and extra carbon sources (different carbon source), etc., and check if one of the two states of a putative switch is enriched within a category of conditions. Using this approach, we correctly uncovered genes that have switch-like behavior, namely GAL1, GAL2, GAL7 and GAL10 (Figure 3-C). These genes all have ΔAIC values that rank among the top 5% and show bimodality, with one of their two modes containing conditions from the same category, i.e. "adding extra carbon sources". The bimodal profiles show that by adding 2% (weight to volume) extra carbon sources into the media, with the exception of galactose as the extra carbon source, the expression of these four genes shut down. It has been reported that these four genes function in the same pathway for galactose utilization, i.e., the well-known "GAL genetic switch" (review: [27]). The addition of alternative carbon sources results in "glucose-repression" of the GAL pathway. During this process, the high level of glucose or other carbon sources (other than galactose) induces the formation of the repressor complex (protein Mig1p and Cyc8-Tup1) and upon its binding to specific upstream repressing sequences (URSG) on the GAL promoters, it prevents the activation of these four GAL genes by the transcription factor GAL4, thereby turning off the galatose utilization pathway.

Current knowledge on the existence and functional machinery of other gene switches is limited. However we show next that by integrating information of the regulatory network and proteomic data, the genes with high ΔAIC obtained from our analysis could be possible switches or at least important genes with respect to the phenotypes. We calculate the ΔAIC values of transcription factors in the yeast transcriptional regulatory network (based on binding motif data, see METHOD), and observe that the leaf-nodes --- genes that are only regulated by one factor and are not regulating any other transcription factors ---- tend to have significantly lower ΔAIC value (average ΔAIC = 135 ± 9 compared with overall average ΔAIC = 223 ± 58 for transcription factors, p < 0.01, also see Additional file 1, Figure S2). These genes which have few regulators and do not transcriptionally control transcription factors are less likely to have feedbacks at the transcriptional level, and therefore switching dynamics. Thus the dynamic property we infer of the molecular components within a network is contingent on the network organization.

Next, we analyze single-cell proteomic data that includes noise in the protein expression measurements. We find a weak but significant negative correlation between the ΔAIC value of a gene and its coefficient of variation, which captures the noise of its protein expression (Figure 3-B and Additional file 1, Figure S3). This suggests that genes with higher ΔAIC value, showing bimodality, tend to express relatively lower levels of noise. This observation that genes with lower expression noise under normal conditions are more tightly controlled highlights their importance in the network, and is consistent with previous suggestions that gene switches have noise-filtering capacity [4,5].

### The Application in Human microarray data: Identifying cancer molecular targets

We further apply the mining approach to an integrated human gene expression dataset (ArrayExpress E-TABM-185 [28]) which collated 5897 microarray experiments performed on the same platform, across a wide range of conditions and cell types, including normal human tissues, carcinoma cells and tissues, hematopoietic cells, and other diseases.

**Figure 4:**
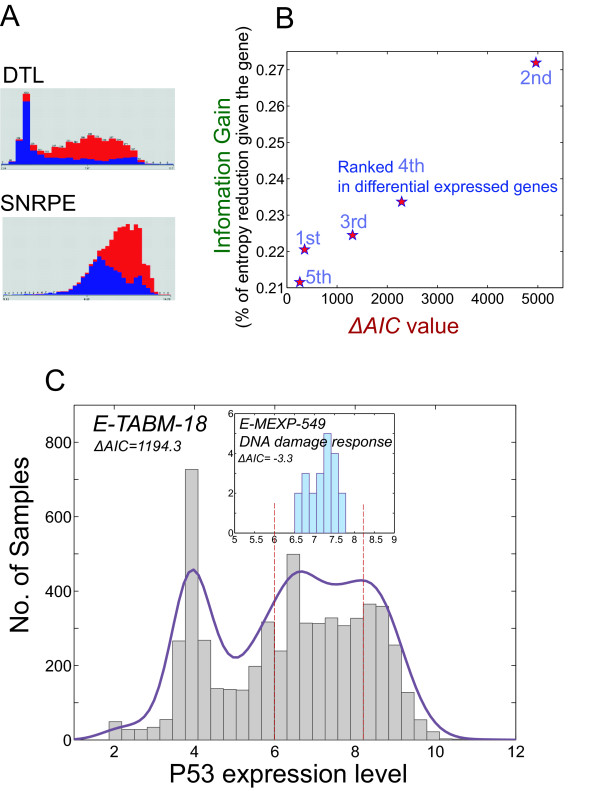
**Application in human microarray dataset**. **A) **Comparison of the differentially expressed genes (with respect to cancer/non-cancer phenotypes) with high and low ΔAIC values. In the histograms, the red bars are cancer samples and the blue bars are non-cancerous samples. **B) **The relationship between information gain and ΔAIC value for the top 5 differentially expressed genes. **C) **A histogram of p53 expression levels in ~6000 sample dataset (E-TABM-18) as compared with a small sample-size dataset (E-MEXP-549, see insert).

A similar ΔAIC distribution (Additional file 1, Figure S4) is obtained where only a few genes show bimodality. For example in Figure 4-A, we compare the histograms of expression levels of two genes, DTL, which is bimodal and ranked 32^nd ^in terms of differential expression, and SNRPE, which is unimodal and ranked 6^th^. DTL, however, shows a bimodal distribution with respect to cancer/noncancer (Figure 4-A) and is more predictive based upon information gain (27% for DTL vs. 25% for SNRPE, see METHOD for the calculation), indicating the prediction of the cancer phenotype based on DTL is slightly better than SNRPE. This is consistent with previous studies where DTL was reported as an essential regulator of the early G2-M checkpoints [29], and assumes important roles in cell cycle progression and differentiation [30]. DTL has been suggested as a gene marker for breast [31] and prostate cancers [32], while the SNRPE gene has not been reported to be associated with cancer. Moreover, among the top 10 most differentially expressed genes for distinguishing the cancer/non-cancer phenotypes, those with higher ΔAIC values provide more information (Additional file 1, Figure S5). Figure 4-B shows the correlations between ΔAIC value and the information gain for the top 5 most differentially expressed genes and suggests that bimodality could be a relevant feature in identifying potential molecular targets.

This large integrated dataset [28] provides a sampling of the state-space of the gene network, and interestingly, the p53 gene (Figure 4-C), reported to be up-regulated in response to DNA damage [33] shows bimodality. This information cannot readily be obtained from comparing the expression data from just two conditions, normal and γ-irradiation [34] for instance (Figure 4-C). Recent single cell measurements with high temporal resolution observed p53 pulses with fixed amplitude and duration, suggesting an on/off rapid switching in the p53 dynamics [35-37]. Although p53 is regulated coordinately on multiple levels (transcription, translation, post-translational modification), our analysis of bimodality provide evidence to support a possible switching dynamics of p53 at the transcriptional level.

### Identify characteristic signatures of human breast cancer

We analyze a paired breast cancer/normal tissue expression dataset (GSE15852) [38] against the integrated human gene expression dataset [28] to identify characteristic signatures of human breast cancer. First, we calculate the separation value D [39] for the top 10% ranked genes by ΔAIC to examine whether the expressions of these genes are bimodal when comparing the breast cancer (1119) samples against all other phenotypes (4,777 samples for ~300 conditions). Biologically this indicates whether a gene potentially shows bistability and could be involved in the "switching" or transition to a breast cancer phenotype. D > 2 has been suggested to indicate whether the separation into two Gaussian distributions or modes is distinctive [39]. Considering the large amount of noise in the microarray data, we accept separation values of greater than or close to 2 (> 1.8) to indicate bimodality, which results in 17 genes showing distinct bimodality in breast cancer.

**Figure 5:**
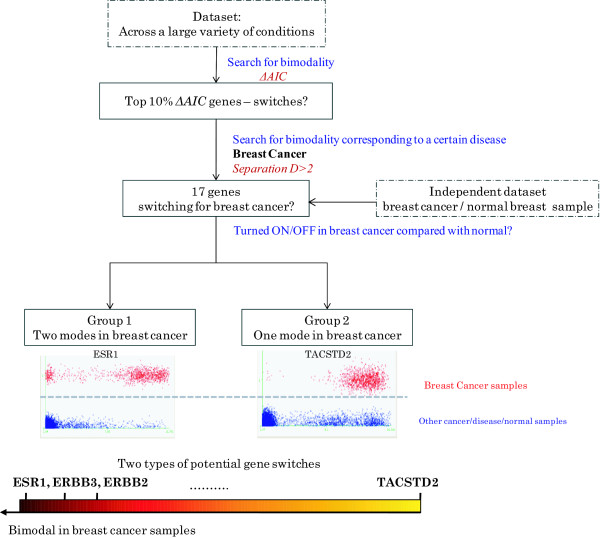
**Identification of potential gene switches for breast cancer**. We analyzed the integrated dataset (E-TABM-185) that contains 5,896 samples from about 300 different conditions to search for bimodality in the gene expression profiles. Genes are ranked based on their ΔAIC calculations, which represent the significance of bimodality in their expression profiles. The top 10%, or about 2000 genes that have the highest ΔAIC values are selected to compute the separation D with respect to breast cancer, among which 17 genes are discovered to express at a distinctive state in breast cancer as compared with all other conditions. An independent dataset (GSE15852, the dotted rectangle box in the Figure) is then used to examine the expression profiles of this 17 genes. The dataset has 43 pairs of samples, each pair consists of a tumor tissue and its adjacent non-tumorous tissue from the same patient. 12 of the 17 genes show different distribution between the breast cancer samples and their paired normal samples. These 12 genes (ESR1, SPDEF, IRX5, ERBB3, ERBB2, CRABP2, RAB25, FXYD3, TACSTD2, DSP, AGR2, CDH1) fell into two types of expression patterns. One type of genes, Type 1, shows bimodality within the breast cancer samples, and they are differentially expressed in some but not all of the paired dataset of breast cancer and normal samples. In other words with Type 1, the normal samples are in the OFF mode while the breast cancer samples contain both ON and OFF states. The other type of gene switch, Type 2, shows predominantly one modality in the breast cancer samples (ON) vs. in normal samples (OFF), thus the genes are differentially expressed in almost all breast cancer/normal pairs.

Next, an independent microarray dataset (GSE15852) with 43 paired breast cancer samples of diverse histopathological characteristics is analyzed to test if the 17 genes are expressed differently and show distinct bimodality in breast tumor as compared to normal breast tissues. Comparing such "local" expression profile (paired normal and cancer conditions) with the "global" expression profile (across various conditions) identified that of these 17 genes, 12 genes (ESR1, SPDEF, IRX5, ERBB3, ERBB2, CRABP2, RAB25, FXYD3, TACSTD2, DSP, AGR2, CDH1) show bimodality in both datasets (Figure 5 shows the flow chart of the procedure). One type of genes is bimodal within the breast cancer samples, herein denoted ***Type 1***, with estrogen receptor-alpha (ESR1) having the highest separation. The other type of gene switch shows predominantly one modality within the breast cancer samples, herein denoted as ***Type 2***, and is where we find the TACSTD2 (a.k.a. Trop2) gene having the highest separation value within this group.

**Figure 6:**
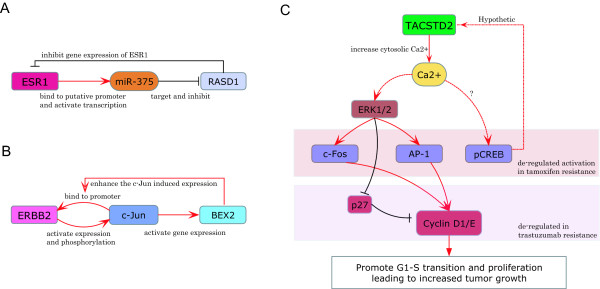
**The potential regulatory mechanism of the identified gene switches for breast cancer**. **A) **ESR1 activates the transcription of a microRNA, miR-375, probably by binding to the putative promoter of the microRNA. miR-375 targets and inhibits the expression of RASD1 (ras dexamethasome-induced 1) gene, which is an inhibitor of the ESR1 gene expression. With this positive feedback, ESR1 can activate its own expression by reducing the inhibition of RASD1 through activation of miR-375. This model was suggested by [42]. **B) **ERBB2 activates the expression and phosphorylation of transcription factor c-Jun, which is able to bind to the promoter of ERBB2 to further induce ERBB2 transcription. This potential positive feedback is likely enhanced through c-Jun dependent activation of BEX2 (brain expressed X-linked 2) gene. This model was suggested by [44]. **C) **A hypothetical regulatory role of TACSTD2 in breast cancer cells. The activation of TACSTD2 increase the cytoplasmic calcium (Ca2+) level, which could in turn activate CREB and the MAPK/ERK pathway through calmodulin-dependent protein kinases (e.g. CaMKII). The activated MAPK pathway can increase the expression of cyclin D1 and cyclin E as well as reduce the level of CDK inhibitor, p27, to thereby promote cell proliferation. The activated CREB could bind to the promoter of TACSTD2, and form a positive feedback to promote and maintain the ON state of TACSTD2. Tamoxifen resistance is associated with the disregulation (high expression level) of c-Fos, AP-1 and pCREB activation [68], which could possibly be mediated by a constitutive ON state of TACSTD2. Trastuzumab resistance is associated with the disregulation of p27 and cyclin D/E (constitutive activation of cyclin D/E and the reason is unclear) [69], which could be modulated by activation of TACSTD2.

Many of the genes that show Type 1 bimodal behavior also exhibit the biomdality within the breast cancer samples (Figure 5). Known therapeutic targets for breast cancer, such as ESR1, ERBB2 (HER2) and ERBB3 (HER3), are identified as showing bimodality in their gene expression level in breast cancer. Their bimodality in the cancer samples represents well-known subtypes in breast cancer, i.e. ER+/ER- and HER2+/HER2- subtypes. ESR1 (estrogen receptor alpha) is a well-known transcription factor involved in the development and progression of breast cancer. Previous immunohistochemical analysis showed a bimodal distribution in estrogen receptors (ER) expression ---- the majority of breast cancer patients express either ER-negative (low expression) or unambiguously ER-positive (high expression), of which (~80%) are ER+, while moderate ER immunostaining is rarely observed [40]. This supports our discovery of bimodality of the ESR1 gene expression within the breast cancer samples. It has been a decade since researchers attempted to explore the mechanism underlying such an all-or-none expression pattern of estrogen receptors. It was previously reported that the ESR promoter activity is increased by co-transfection of the wild-type ESR expression vector, suggesting a positive contribution of ESR to its own expression [41]. A recent study uncovered that miR-375 is involved in a forward feedback loop that regulates ESR1 expression, whereby ESR1 enhances miR-375 expression and miR-375 targets and reduces the expression level of RASD1 (ras dexamethasone-induced 1) gene, which is a transcriptional inhibitor of ESR1 [42]. These studies provide evidence of a potential positive-feedback (with a double-negative circuit) induced bistability of the ESR1 expression, as shown in Figure 6-A, where the topology is similar to the toggle-switch design in Figure 1-D. ERBB2 and ERBB3 interact with each other and are known to be transcriptionally regulated by ESR1 [43]. A recent study [44] identified a positive feedback of ERBB2 through the transcription factor c-Jun, which could provide a potential explanation for the bimodality observed for ERBB2, as shown in Figure 6-B.

The molecular characterization of the Type 1 genes (e.g. ESR, HER2) suggests the development of therapies for ER+/PR+ and HER2+ would be effective for these breast cancer subtypes, however ~15-20% of the breast cancer tissues expressing low levels of these biomarkers (i.e. triple negative subtype) have poor prognosis and few treatment options. Moreover, patients that are responsive to commonly used drugs, such as tamoxifen (estrogen antagonist) and trastuzumab (anti-HER2 agent), eventually acquire resistance to the drugs. ~30% of tamoxifen-responsive tumors become resistant [45,46], and the resistance invariably ensues at some point with trastuzumab. Given the increase in resistance to drugs that target the ESR receptor alternative therapeutic targets are needed.

**Figure 7:**
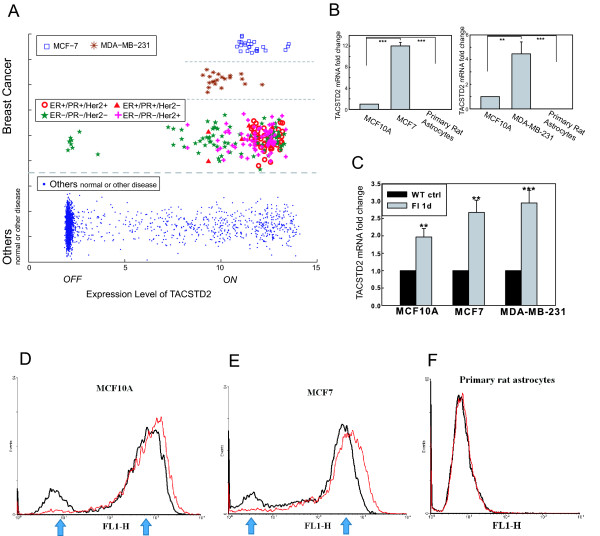
**The switch-like behavior of TACSTD2 in breast cancer and its regulation**. **A) **The scatter plot shows the gene expression level of TACSTD2 in the samples of breast cancer and other phenotypes from the integrated microarray dataset (E-TABM-185). Each datapoint in the scatter plot represents the TACSTD2 expression in one of the samples, with the x-axis indicating the expression level (the values are Log2(microarray-Signal)). In order to show all the samples, the values in y-axis are randomly generated to reduce the overlap between samples with similar TACSTD2 expression level. The breast cancer samples and the breast cancer cell lines, MCF-7 and MDA-MB-231, are separated from other samples for better comparison. Subtypes of breast cancers are determined by their expression levels of ESR1, PR, and Her2. Overall TACSTD2 is in the ON state for 99% of all breast cancer samples in the dataset (Additional file 1, Table S3). **B) **The TACSTD2 mRNA levels in human mammary epithelial cell line, MCF10A, in breast cancer cell lines, MCF7 and MDA-MB-231, and in primary rat astrocytes were measured by quantitative real-time PCR (n = 3). **: p < 0.01, ***: p < 0.001. A line indicates comparison between the two bars connected by the line. **C) **The mRNA-fold change of TACSTD2 in human mammary epithelial cell line and the different breast cancer cell lines upon FI treatment. Quantitative real-time PCR was performed to measure TACSTD2 mRNA expression levels in MCF10A, MCF7, and MDA-MB-231. The untreated cells (controls) and cells treated with 10 μM forskolin and 100 μM IBMX (FI) for 1 day (n = 3) are shown. **: p < 0.01, ***: p < 0.001. **D), E), F) **Flow cytometry analysis of TACSTD2 expression in MCF10A, MCF7 and primary rat astrocytes (Black lines). The cells were treated with 10 μM forskolin and 100 μM IBMX (FI) for 1 day (Red lines) and the two modes of TACSTD2 in MCF10A, MCF7, and primary astrocyte cell population are pointed out by the blue arrows. Note the primary astrocytes have only one mode.

The second type of potential gene switch, herein denoted as Type 2, shows unimodal behavior in the breast cancer tissue (Figure 5) and is differentially expressed in almost all the paired breast tumor/normal tissues as compared with non-breast cancer samples. The top gene showing this type of switching behavior is TACSTD2 (tumor-associated calcium signal transducer, also known as Trop2). Type 2 gene switches uncovered by our analysis show a distinct state in the breast cancer samples, and could be a potential biomarker or drug target that does not rely on the ESR receptor. We characterized the TACSTD2 gene, and found it to be distinctively expressed at higher levels in almost all of the breast cancer samples, ER+/-, HER2+/- subtypes (Figure 7-A). We confirm that the expression of TACSTD2 gene is high in breast cancer cell lines MCF7 and MDA-MB-231 (Figure 7-B) as compared with non-cancer cells (i.e. primary rat astrocyte).

**Figure 8:**
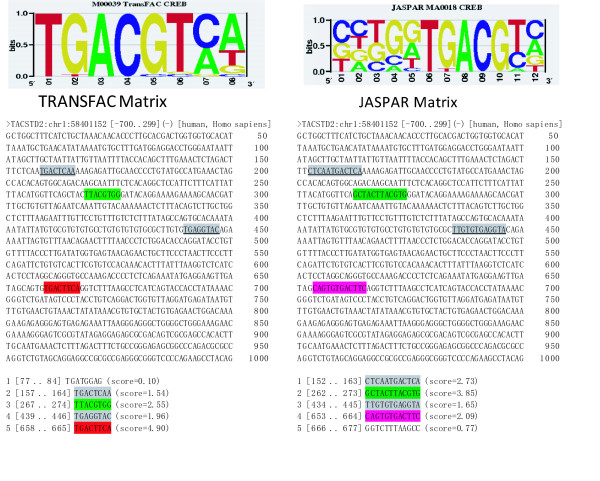
**CREB binding sites on the promoter of TACSTD2**. We extracted the promoter sequence of TACSTDs from TRED (the Transcriptional Regulatory Element Database, http://rulai.cshl.edu/TRED), and searched for the CREB binding sites by comparing the promoter sequences with the position weight matrix (PWM). The TRANSFAC http://www.gene-regulation.com and the JASPAR http://jaspar.genereg.net/ databases provide different versions of CREB binding PWM. Nevertheless, there are four potential CREB binding sites that are predicted by both of the PWMs on the promoter of TACSTD2.

Currently little is known about the regulation of TACSTD2. Promoter analysis (Figure 8) identified CREB as a potential transcription factor that regulates the expression of TACSTD2. We observe a significant increase in the correlation between the expression level of CREB and TACSTD2 in the breast cancer samples. The correlation coefficients in the normal breast tissue are 0.15, 0.06, 0.03 for the three CREB probes in the Affymetrix array, and the correlation coefficients in the breast tumor tissues are 0.46, 0.21, 0.31, respectively, suggesting CREB could regulate TACSTD2.

To assess the possible switching behavior and regulation by CREB of TACSTD2, we performed flow cytometry to probe the TACSTD2 protein level at single-cell resolution. For both MCF10A and MCF7 breast cell lines the TACSTD2 protein level shows a bimodal distribution in their cell population (Figure 7-D E F), which is a property of a bistable system. We stimulated the cells with FI (Forskolin and IBMX) to induce cAMP, which is an activator of CREB [47], and measured the TACSTD2 levels. Both TACSTD2 mRNA and protein levels increased significantly upon stimulation (Figure 7-C, and Figures 7-D, E, respectively; for quantification of flow cytometry results see Additional file 1, Table S2), thereby supporting a possible transcriptional regulation by CREB. Upon activation of TACSTD2 by FI, a decrease in one of the modes with a concomitant increase in the other mode, instead of a gradual increase in the protein level, (Figure 7-DE) is indicative of a switching behavior. The activation essentially increases the number of cells with TACSTD2 levels at the ON state and decreases the cells with TACSTD2 at the OFF level. In contrast, the expression of the TACSTD2 protein in primary rat astrocytes shows a unimodal expression under the same test conditions. Furthermore stimulation of astrocytes by FI leads to a non-significant change in the protein level and with the cells predominantly remaining in the OFF steady-states (Figure 7-F).

The activation of TACSTD2 has been suggested to transduce calcium signal, likely by mediating calcium release from intracellular stores [48]. It has been shown that the cross-linking (stimulation) of the TACSTD2 gene leads to a significant rise in the cytoplasmic calcium (Ca2+) level [48]. The release of calcium can activate CREB [49] and the MAPK/ERK pathway [50] through calmodulin-dependent protein kinases (e.g. CaMKII). Indeed it is reported [51] in murine system that a high level of TACSTD2 can activate MAPK signaling to induce c-Fos and AP-1. This results in elevated levels of CycD1 and CycE as well as reduced levels of the CDK inhibitor, p27, which together can de-regulate and promote cell proliferation [51].

In light of these studies, our analysis uncovered TACSTD2 gene to have a switch-like behavior, with CREB as a possible regulator, which is activated in breast cancer and modulates the transcription of TACSTD2. CREB can provide a positive feedback in the transcriptional regulation of TACSTD2 (Figure 6-C), to thereby support a bimodal distribution in TACSTD2 expression and possible bistable behavior.

## Discussion

### Mining approach to identify gene switches

Researchers recognized that "genetic switches" behave in a discrete manner, but this feature is usually lost in biochemical analysis of large cell populations due to the difficulty in distinguishing between changes in the proportion of cells and their expression level in the two states [9]. For example, it is hard to determine from population measurements whether the expression level of a gene increases gradually by 70%, or whether 70% of the cells are "switched" ON. In this study, we provide an alternative approach to identifying possible gene switches by capitalizing upon the large amount of available microarray data. The large sample set enables the characterization of the state space by uncovering the presence of the two attractor-states where the majority of the samples should fall. Thus, if an ON/OFF switch behavior exists in a system the state space will show bimodality or bistability, which are relatively stable with respect to perturbations [52]. It has been suggested that bistability or multiple steady states [23] exists in large gene networks [53,54], and these attractor-states represent different phenotypes [23]. Thus, by sampling across different conditions, which are less affected by population averaging, one can reveal this dynamic feature of regulatory networks.

Our mining approach demonstrates that in the absence of *a priori *knowledge of the specific network architecture, one can capitalize on genome-wide expression profiling to capture dynamic properties of a complex network.

### Meta-analysis of expression data

The increase in publically available microarray repositories provides a tremendous potential for data mining to unravel knowledge of cellular processes. Current approaches that integrate and analyze the wealth of expression data continues to emerge. The concept of "meta-analysis" comes from statistics and has been extended to integrate analysis of expression data. However most of the current studies have focused on database comparison, integration, and clustering [55]. Furthermore, the statistical analysis of combining datasets of differentially expressed genes [56-58] have been used primarily to enhance the statistical power, i.e. reducing false discovery rate, as oppose to providing insight into the biological mechanisms.

Our study provides a different perspective that takes advantage of the large integrated set of expression data, and suggests a mechanism-based framework to perform the meta-analysis. This approach of integrating microarray data from a diverse set of conditions provides a common "context" of gene behaviors, whereby one can obtain a better understanding of the specific function of a gene for a particular condition under investigation. The example of p53 expression, shown in Figure 4-C is a case in point. p53 is known to be a major regulator in response to DNA damage [33], however it is difficult to identify from a small set of microarray experiment (E-MEXP-549, 21 samples, collected under the condition of DNA damage response) since it does not appear in the top ranked differentially expressed genes ([34], http://www.ebi.ac.uk/gxa/experiment/E-MEXP-549). This is likely due to its multi-level regulation (transcription, translation, post-translational modification) and also the lack of appropriate control conditions in the experiment. Nevertheless, by comparing the small set of samples against the global expression of the integrated dataset provides a "context", whereby one observes a significant reduction in the variance in expression within the small set of microarray experiment (E-MEXP-549), and suggests tightly-controlled regulation of this gene in DNA damage response.

Although p53 has both switching and oscillation dynamic features [35-37], we only discovered and discussed its switching property with our novel approach. Our approach identifies switch-like behavior based on the bimodal distribution induced by the feature of bistability. Oscillatory dynamics could have multiple (more than two) steady states and furthermore, the cells in the microarray experiments are not necessarily synchronized according to the periodic feature of the oscillation dynamics being investigated, thereby making it difficult to uncover this type of dynamics. Our approach is designed to discover gene switches and currently cannot be directly apply to identify oscillatory dynamics.

### TACSTD2 is an attractive candidate for drug therapy of breast cancer

Our mining approach uncovered a unique expression pattern of TACSTD2 in breast cancer, and experiments confirmed TACSTD2 show bimodal behavior in breast cancer cell lines. TACSTD2 (Trop2) is a cell surface glycoprotein, first discovered to be highly expressed in trophoblast cells that become invasive and metastasized to form the outer layer of blastocyst in embryo development [59]. Recent studies, along with our analysis of breast cancer samples, found TACSTD2 to be highly expressed in a variety of epithelial cancers and show low to no expression in normal somatic cells. High expression of TACSTD2 in squamous-cell carcinoma [60], pancreatic [61], colorectal [62] and gastric [63] cancers have been associated with poor prognosis and higher incidence of metastasis and death. TACSTD2 was identified as an oncogene in colorectal cancer cells [64]. Although not essential for cell proliferation under normal condition, ectopic expression of TACSTD2 enhances anchorage-independent cell growth, promotes tumorigenesis and metastasis in colon cancer cells. Knock-down or inhibition of the protein reduces the invasiveness of aggressive colon cancer cells [64]. In our analysis we also found TACSTD2 to be highly expressed in many colon cancer samples and shows bimodality (Additional file 1, Figure S6), however the percentage of colon cancer samples with TACSTD2 at the ON state (~60%) are less than in breast cancer (~99%), suggesting TACSTD2 could be a better target for breast cancer.

In previous microarray analysis of breast tumors, [65] Huang et al. studied "aggregate patterns of gene expression" with respect to lymph node status and recurrence, and identified "metagenes" that could predict the outcomes of the patients. TACSTD2 is found among the metagenes in their list; however the list consists of more than a hundred genes with potential predictive value. In contrast, we find the TACSTD2 gene to be the top gene in the list (Additional file 1, Table S3) that shows the Type 2 behavior. Interestingly, the distinctive HIGH/LOW expression level of the TACSTD2 gene has been implicated as a marker for stem cell characteristics in prostate basal cells [66] and hepatic oval cells [67]. The prostate basal cells and hepatic oval cells, considered progenitor cells, show HIGH expression of the TACSTD2 gene and maintain self-renewal capability [66,67], and thereby implicating a potential role of TACSTD2 in cancer initiating stem cells.

Although TACSTD2 has been reported to be associated with cancer, the regulatory mechanism of TACSTD2 remains unclear. Combining computational prediction and experimental analysis, we found that CREB could regulate TACSTD2 in breast cancer cells, and suggest a potential feedback structure of TACSTD2 regulation (Figure 6-C). To the best of our knowledge, this is the first regulatory circuit discovered to control TACSTD2 expression.

Studies of tamoxifen-resistant breast cancer cells found these cells develop altered activation of CREB and AP-1 [68], which we speculate could be related to TACSTD2 signaling. Trastuzumab resistance in HER2+ breast cancer cells is reported to involve elevated CycE expression level which is associated with the desensitization of p27 regulation [69]. Given that TACSTD2 could increase CycE level by inhibiting p27, it provides a possible mechanistic connection with the TACSTD2 gene as a potential target for ERBB2/HER2 regulation and trastuzumab resistance (Figure 6-C).

Overall, our computational analysis demonstrate a distinctively high expression of TACSTD2 in almost all ER+/-, HER2+/- subtypes of breast cancer. Experiment shows that TACSTD2 expression is high in breast cancer cell lines, MCF-7 and MDA-MB-231 (Figure 7-B), and FI treatment enhances the expression of TACSTD2 (Figure 7-C and Additional file 1, Table S2). Comparing MCF-7 (an ER+/ERBB- cell line) and MDA-MB-231 (a triple negative cell line) cells, many more cells in the MDA-MB-231 than in the MCF-7 cell line have TACSTD2 in the ON state (Additional file 1, Table S2 and Figure S7). In fact most cells in the MDA-MB-231 cell line have TACSTD2 turned ON. This observation, together with the information extracted from the microarray data (Figure 7-A), highlights TACSTD2 as an important biomarker for both ER+ and ER- breast cancer subtypes, as well as an attractive candidate for drug therapy against the triple negative (ER-, PR- (progesterone receptor) and HER2-) subtype of breast cancer, with potential implications for treating drug-resistant cases that are non-responsive to ER/HER2-targeted therapies. In addition, the presence of TACSTD2 on the cell surface makes it more accessible to antibody-based therapeutics.

## Conclusions

We propose a top-down mining approach to exploring gene switches on a genome-scale level. Our mining approach demonstrates that in the absence of *a priori *knowledge of the specific network architecture, one can capitalize on genome-wide expression profiling to capture dynamic properties of a complex network. To the best of our knowledge, this is the first attempt in applying mining approaches to explore gene switches on a genome-scale. By applying the computational analysis on human microarray data, we uncovered a unique expression pattern of TACSTD2 in breast cancer, and experiments confirmed TACSTD2 show bimodal behavior in breast cancer cell lines, further, our perturbation study suggest a potential bistable mechanism is involved. To the best of our knowledge, this is a first case a single cell level bimodality and bistability can be predicted from microarray data. Combining our computational and experimental analysis, together with previous studies in the literature, we suggest TACSTD2 could be an attractive candidate for drug therapy against both ER+ and ER- subtypes, including possibly the triple negative (ER-, PR- (progesterone receptor) and HER2-) subtype of breast cancer, and finally with potential implications for treating drug-resistant cases that are non-responsive to ER/HER2-targeted therapies.

## Methods

### 1. Kinetic models and simulation

The ordinary differential equations (ODEs) for synthetic system in Figure 1-A are as follows:

$$\frac{\text{dA}}{\text{dt}}=p\left[\frac{A^{2}}{1+A^{2}}\right]\left[\frac{1}{1+R^{2}}\right]\left[1-\frac{A}{2.5}\right]-\text{deg}\left(A\right)$$

$$R\left(i\right)=10\left[1-\frac{K_{i}}{1+K_{i}}\right]$$

*A *represents the expression level of Gene A, *pA^2^/[1+A^2^] *describes the self-binding and activation of the transcription, and *1/1+R^2 ^*is the effect of the repressor, in which R depends on the stimulation *i*--the concentration of the inhibitor *i*. *deg(A) *is a linear function for the degradation of *A*. The model is constructed by [24] for a mammalian cell system.

The ordinary differential equations for synthetic system in Figure 1-D are as follows:

$$\frac{dA}{dt}=\frac{a}{1+B^{2}}-\text{deg}\left(A\right)$$

$$\frac{dB}{dt}=\frac{a}{1+A^{2}}-\text{deg}\left(B\right)$$

*A, B *represents the expression level of Gene A and B, respectively. *α *is a parameter about the strength of the cross-repression of the two genes. *deg() *is a linear function for degradation. The model is constructed by [16] in *E.Coli*.

The kinetic equations and parameters for the E2F-Rb system is obtained from the previous study by Yao et al. [15].

ODEs are implemented in MATLAB and numerically simulated with Runge-Kutta Method. Multiple simulations with different initial conditions are performed with customized MATLAB codes.

### 2. Quantifying the bimodality

Usually researchers use the DIP statistics [70] or Gaussian mixture model to [25]http://www.astro.lsa.umich.edu/~ognedin/gmm/ identify bimodal distributions. The DIP method provides a statistical test for uniform distribution (the null distribution is uniform; data distribution is estimated by empirical kernel function and compared with the null distribution). But what is needed is not only a quantity for bimodality, but also an explicit separation of the conditions into two categories corresponding to two expression levels, thus we choose the Gaussian mixture model. The Expectation Maximization algorithm is implemented to separate the data distribution into two Gaussian models. We compare the fit of the data to the two Gaussian models vs. the one Gaussian model to see if the distribution is bimodal. The criterion used to assess the fitting is the Akaike information criterion from information theory.

$$AIC=2k-2log\left(L\right)$$

Where k represents the number of parameters, and L is the goodness of fit, defined by the likelihood of observing the data given the model (one or two Gaussian in this case). The lower the AIC value, the better the model fits the data. Thus we defined a "ΔAIC " value as:

$$\Delta AIC=AIC_{1}-AIC_{2}$$

in which *AIC_1 _*is the AIC of the gene expression profile assuming a unimodal distribution, while *AIC_2 _*assumes the profile is a bimodal distribution. ΔAIC compares the fit with a unimodal vs. a bimodal distribution. The higher the ΔAIC value, the more likely the expression profile shows bimodality. Comparing with simpler criteria such as separation and kurtosis, ΔAIC is more reliable, and is more resistance to noise in data (see Additional file 1, Figure S1).

ΔAIC provides a measure for an "unconditional" bimodality in which the profiles show bimodal but the condition for the "switch" is yet to be investigated. When a particular condition is specified (e.g. "the breast cancer phenotype"), the separation D can be used to identify if there is a distinct state for the condition, or, a "conditional specific" bimodality:

$$\text{D}=\frac{|\mu_{1}-\mu_{2}|}{\sqrt{\frac{\sigma_{1}^{2}+\sigma_{2}^{2}}{2}}}$$

Where (*μ_1_, σ_1_*) are the mean and deviation of samples in the specified condition, while (*μ_2_, σ_2_*) are the mean and deviation for all other samples.

### 3. Datasets

The "Mega Yeast Gene Expression Data" is downloaded from http://gasch.genetics.wisc.edu/datasets.html. The dataset contains 500 yeast microarray experiments. The conditions include environmental stress [71], cell cycle [72], sporulation and various other perturbations. A yeast putative transcriptional regulatory network based on the known motifs on the gene promoters is obtained from the YEASTRACT database [73]. Information on the noise of the yeast protein expression is extracted from the Integrate Single-cell Proteomic Analysis data [74].

The human gene expression dataset (ArrayExpress E-TABM-185) is a per platform integration provided by ArrayExpress database http://www.ebi.ac.uk/arrayexpress/. The dataset integrates (and normalized) 5897 microarray experiments performed on the same Affymetrix GeneChip Human Genome HG-U133A platform [28]. A variety of conditions and cell types are involved, including normal human tissues, carcinoma cells and tissues, hematopoietic cells, Alzheimer's disease, asthma, Down syndrome, Huntington's Disease, etc. Although it is an integration of experiments from different sources (different labs), the providers have cleared and normalized the dataset such that "the biological signal in these data is significantly stronger than the laboratory effects" [28].

### 4. Calculating information gain to identify prediction value of genes

Based on information theory, we apply Shannon Entropy to represent the uncertainty of the phenotypes, which is defined as:

$$H\left(X\right)=-\overset{n}{\underset{i=1}{\sum }}p\left(x_{i}\right)\;\text{log}\;p\left(x_{i}\right)$$

where X is the sample set (dataset with different phenotypes), p(xi) is the probability (calculated by frequency) that the sample exhibits a particular phenotype or category of phenotypes. Conditional Entropy is then calculated to identify the uncertainty retained given the extra information, i.e. the expression level of a gene:

$$H\left(X|Y\right)=\underset{y\in Y}{\sum }p\left(y\right)H\left(X|Y=y\right)$$

$$=\underset{x\in X}{\sum }\underset{y\in Y}{\sum }p\left(x,y\right)\;\text{log}\frac{p\left(y\right)}{p\left(x,y\right)}$$

Here Y represents the expression level of a gene. Since the expression level is continuous, we discretize this attribute into several categories with equal frequency. Choosing different number of categories in the discretization changes the entropy value in the calculation but does not affect the comparison made in estimating the contribution of genes to the prediction of the phenotypes.

Thus, the entropy reduction (or percentage of information gain) can be defined as

$$\frac{H\left(X\right)-H\left(X|Y\right)}{H\left(X\right)}\%$$

which represents the contribution of gene Y to the prediction of the phenotype (defined by the reduction in the uncertainties given the information of gene Y).

### 5. Experimental studies of TACSTD2

#### Cell culture and materials

Human mammary epithelial and breast cancer cell lines were obtained from Dr. Kathleen Gallo in Michigan State University. MCF7 and MDA-MB-231 were cultured in Dulbecco's modified Eagles's media (DMEM, Gibco BRL, Paisley, PA, USA) with 10% fetal bovine serum (FBS), 2 mM glutamine and penicillin/streptomycin. MCF10A cells were cultured in DMEM/F12(1:1) with 5% horse serum, 10 ug/ml insulin, 20 ng/ml EGF, 100 ng/ml choleratoxin, 0.5 ug/ml hydrocortisone and pen/strep. Primary astrocytes were maintained in DMEM/F12 (1:1) plus 10% FBS and pen/strep. Cells were maintained at 37°C and 10% CO2. Forskolin (Sigma, St. Louis, MO, USA) and isobutylmethylxanthine (IBMX) (Sigma) were used at the concentrations of 10 M and 100 M, respectively.

#### Quantitative real time polymerase chain reaction

mRNA was extracted using the RNA extraction kit (Qiagen, Valencia, CA, USA), then mRNA was reverse transcribed into cDNA using the cDNA synthesis kit (Bio-Rad, Hercules, CA, USA). The following primer sets (Operon, Huntsville, AL, USA) were used for PCR: human-TROP2 (5'- GAGATTCCCCCGAAGTTCTC -3',5'- AACTCCCCCAGTTCCTTGAT -3'), rat-TROP2 (5'-TACTGCTACTGCTGGCGATC-3',5'-GCAGGCACTTGGAAGTTAGC-3'), rat-actin (5'-CTCTTCCAGCCTTCCTTCCT-3', 5'-AATGCCTGGGTACATGGTG-3'), human-actin (5'- TGGACTTCGAGCAAGAGATG -3', 5'- AGGAAGGAAGGCTGGAAGAG -3'). Amplifications of the cDNA templates were detected by SYBR Green Supermix (Bio-Rad) using Real-Time PCR Detection System (Bio-Rad). The cycle threshold values were determined by the MyIQ software.

#### Flow cytometry

Cells were washed with PBS and collected by trypsinization. Cells were then incubated with TACSTD2 primary antibody (BD bioscience, CA, USA; Santa Cruze, CA, USA) at 4°C for 30 min. After washing twice with wash buffer, the cells were incubated with Alexa Fluor-488 conjugated goat anti-mouse secondary antibody for 30 min at 4°C in the dark. Cells were washed twice with wash buffer and resuspended in staining buffer, and then subjected to flow cytometry analysis by BD FACSVantage.

#### Statistical analysis

All experiments were performed at least three times, the results were shown as mean ± standard deviation, and representative results are shown. Statistical analysis were performed by an unpaired, two tail student t-test. * indicates p < 0.05, ** indicates p < 0.01 and *** indicates p < 0.001.

## Authors' contributions

MW and CC conceived of the study. MW carried out the computational analysis, participated in the design of the experimental studies and drafted the manuscript. LL participated in the design of the experimental studies and carried out the experimental studies. CC participated in the overall design and coordination of the study and helped to draft the manuscript. All authors read and approved the final manuscript.

## Supplementary Material

Additional file 1 **Supplementary Tables and Figures**. Supplementary Tables S1-S3 and Supplementary Figures S1-S7. Click here for file
