# Supplementary material for: Identification of novel targets for breast cancer by exploring gene switches on a genome scale
Source: BMC Genomics. 2011 Nov 3;12:547. doi: 10.1186/1471-2164-12-547 (PMC3269833; doi:10.1186/1471-2164-12-547)
Supplement: Additional file 1 — Supplementary Tables and Figures. Supplementary Tables S1-S3 and Supplementary Figures S1-S7. [file 1471-2164-12-547-S1.DOC]

**Supplementary materials**

| **Sample size** | **Top 50 ΔAIC**  **# Genes appeared in both independent sampling** |
| --- | --- |
| 50 | ~22 (44%) |
| 100 | ~32 (64%) |
| 250 | ~40 (80%) |

**Supplementary Table S1.** ΔAIC values calculated based upon two independent random sampling and their rankings were compared. The percentages of common genes ranked in among the top 50 ΔAIC values in both samples are computed. Large sample size provides more robust and less biased ΔAIC rankings.

| **Cell type** | **TACSTD2 expression** | |
| --- | --- | --- |
|  | **untreated (% stained)** | **FI 1d treatment (% stained)** |
| MCF10A | 83.7±1.10 | 97.31±0.11** |
| MCF7 | 91.61±4.8 | 99.26±0.36* |
| MDA-MB-231 | 95.56±1.15 | 98.32±1.28* |
| Primary rat astrocytes | 0.65±0.54 | 0.77±0.60 |

**Supplementary Table S2.** TACSTD2 expression upon FI treatment in different cell types, the quantification of flow cytometry histogram.

* P<0.05; ** P<0.01

| **Gene Symbol** | **Approximate percentage (number of samples) of breast cancer samples with the gene at its ON state** |
| --- | --- |
| **TACSTD2** | **~99% (~1108)** |
| **DSP** | **~98% (~1097-1099)** |
| **ERBB3** | **~96% (~1080-1085)** |
| **FXYD3** | **~96% (~1072-1081)** |
| **RAB25** | **~95% (~1061-1070)** |
| **CDH1** | **~94% (~1055-1063)** |
| **CRABP2** | **~92% (~1018-1041)** |
| **AGR2** | **~90% (~1002-1015)** |
| **SPDEF** | **~90% (~995-1012)** |
| **IRX5** | **~89% (~980-1018)** |
| **ESR1** | **~80% (~887-902)** |
| **ERBB2** | **~65% (~690-791)** |

**Supplementary Table S3. List of gene switches in breast cancer**

The total number of breast cancer samples in the integrated dataset is 1119. The threshold (cutoff) for the ON and OFF states of each gene is approximated by the lower point between the two modes of expression profiles in the bimodal histogram distribution of all the samples. The threshold usually falls at an expression value between 4-6 (after the log2 transformation).

***Note***: For ER, HER2(ERBB2) and PR(progesterone receptor, not actually a switch), the high and low expression of these three genes could determine different subtypes of breast cancer. The cutoff thresholds for positive and negative subtypes were defined as ESR1(9,6)/Her2(10,8)/PR(5,4) by analyzing the expression profiles. For example, the distribution of Her2 expression in all the samples shows a peak at around 7-8 and another peak around 10, so the samples with Her2>10 are referred to as Her2+, while the samples with Her2<8 are denoted as Her2-, and we did not include the samples between 8 and 10 in **Figure 7** of the manuscript since their Her2 subtype are ambiguous.


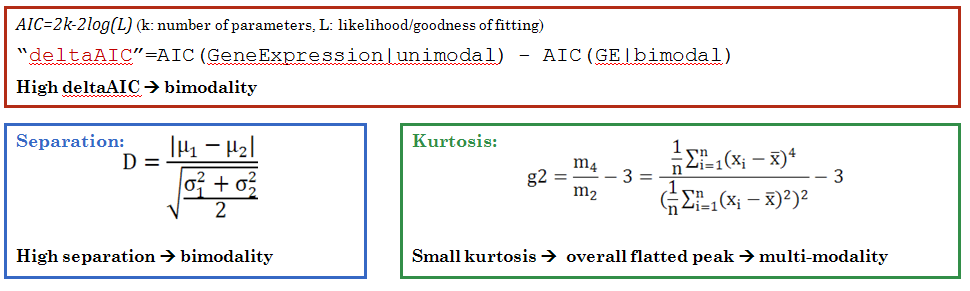

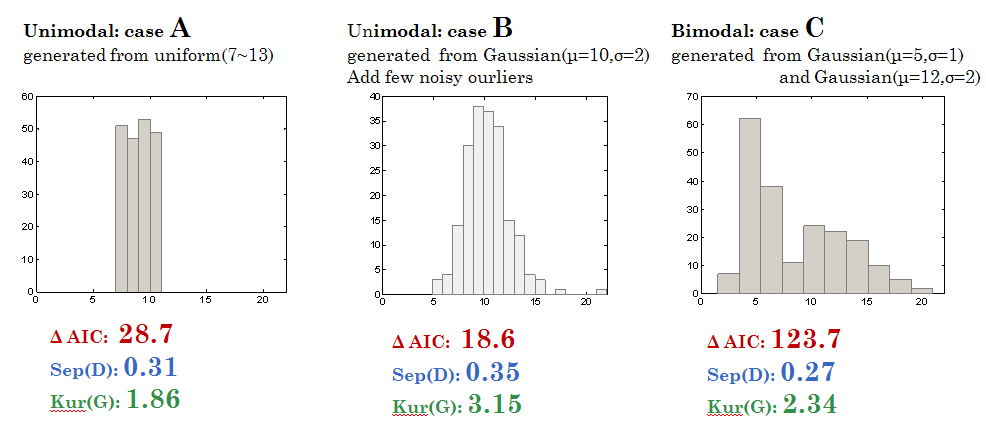
**Supplementary Figure S1.** Definition and comparison of different criteria. ΔAIC values have large differences between unimodal and bimodal distributions, and can be used to distinguish the two distributions. *Kurtosis* is sensitive to the shape of the distribution. Comparing case A with C, *kurtosis* indicates case A is more likely to be bimodal, which is incorrect. *Separation* is sensitive to both the shape of the distribution, e.g. comparing case A with C, and the noise/outliers in the data, e.g. comparing case B with C.


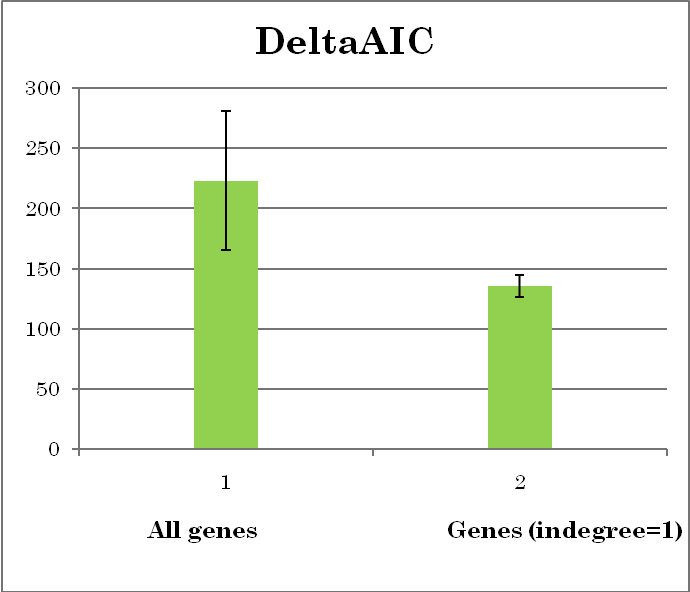


**Supplementary Figure S2.** A comparison of the average ΔAIC of all transcriptional factors with that of the leaf nodes (genes that are only regulated by one factor and are not regulating other transcription factors) in the transcriptional regulatory network.


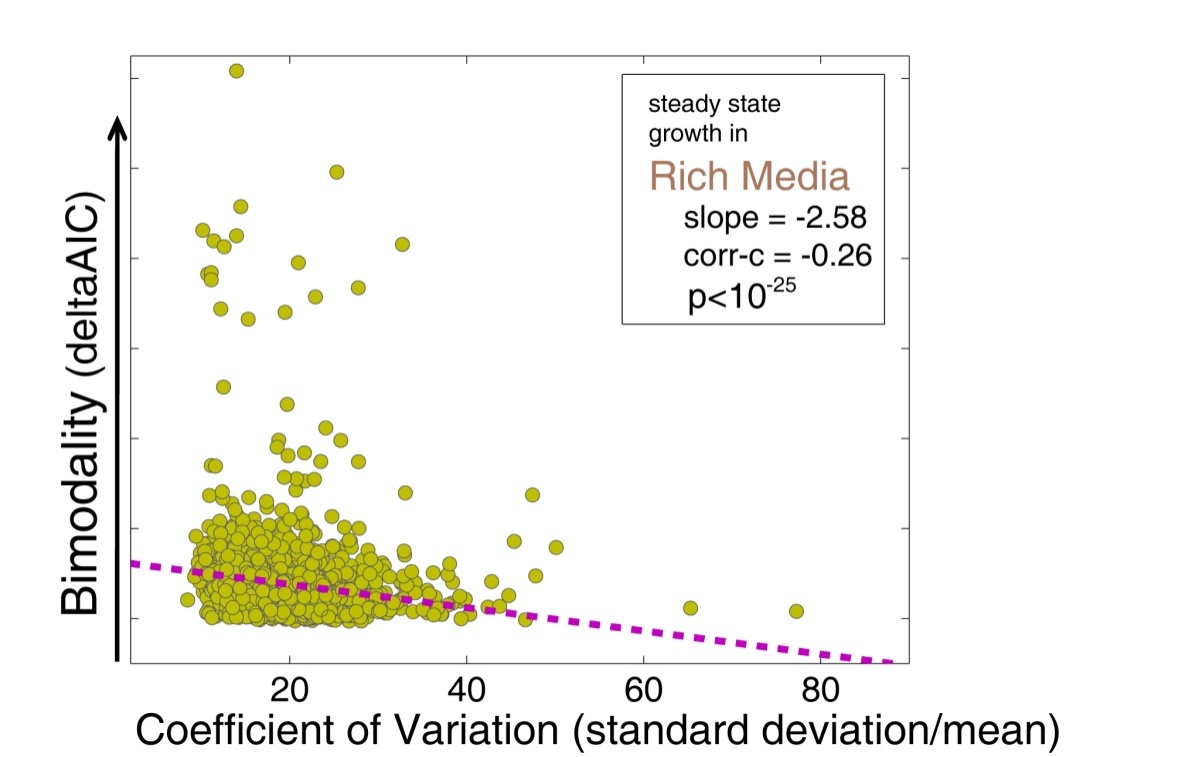


**Supplementary Figure S3.** A negative correlation between bimodality (as defined by ΔAIC) and expression noise (as defined by the coefficient of expression variation) in the yeast genes. (Rich Media, normal condition (YPD)).


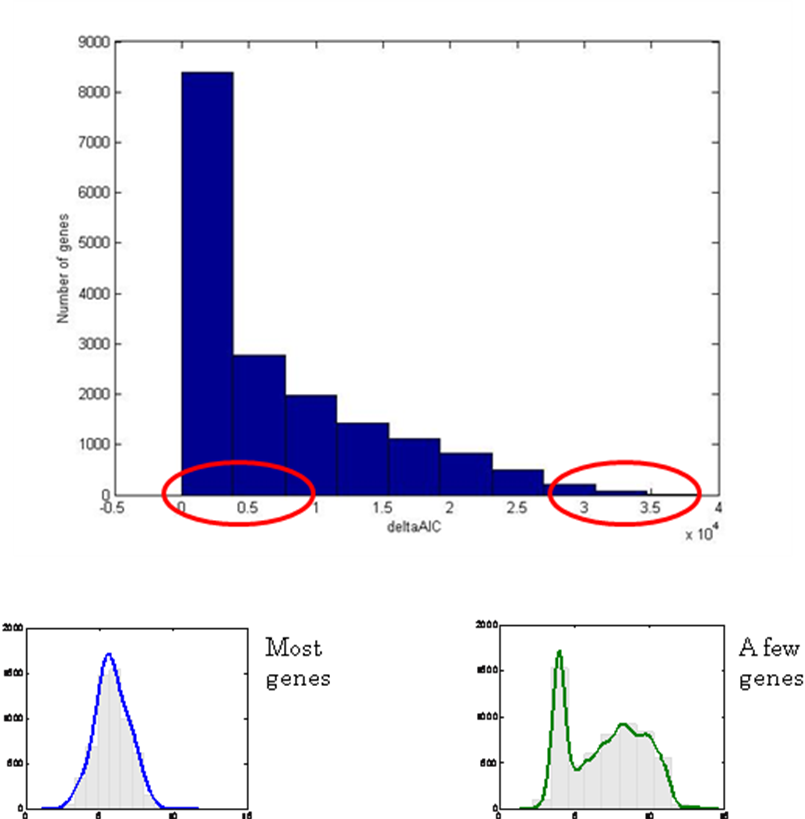


**Supplementary Figure S4.** A histogram of the ΔAIC values among the human genes. Most genes have small ΔAIC and exhibit a uni-modal expression profile. A relatively small number of genes have high ΔAIC and show bimodality in expression.


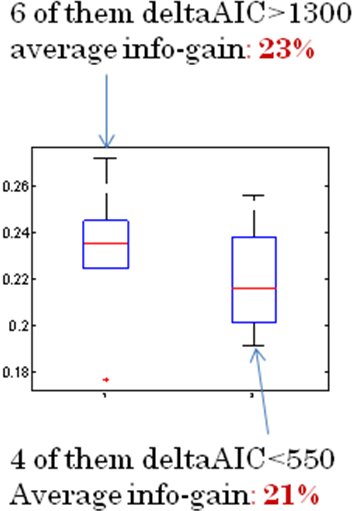


**Supplementary Figure S5.** The relationship between ΔAIC and information gain for the top 10 most differentially expressed genes with respect to the phenotypes: cancer vs. noncancer (based on p-value with t-test).


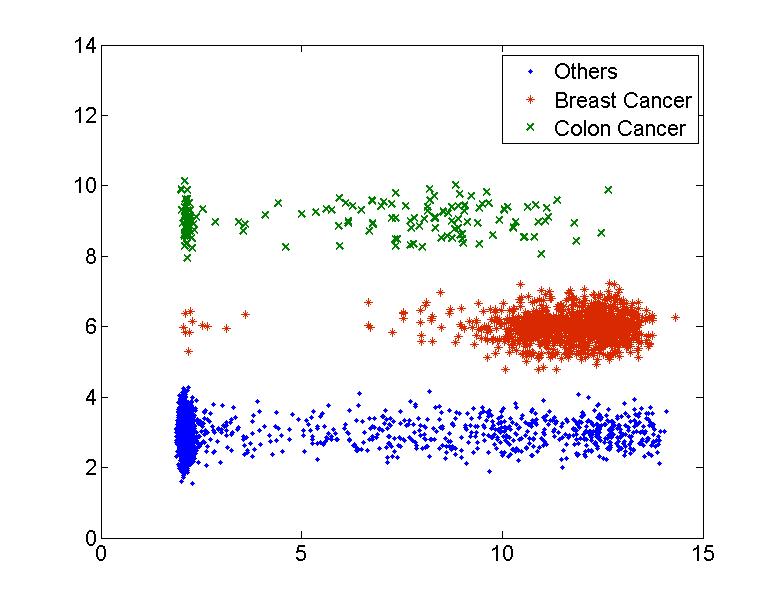


**Supplementary Figure S6.** The scatter plot shows the gene expression level of TACSTD2 in the samples of breast cancer, colon cancer, and other phenotypes from the integrated microarray dataset (E-TABM-185). Each datapoint in the scatter plot represents the TACSTD2 expression in one of the samples, with the x-axis indicating the expression level (the values are Log2(microarray-Signal)). In order to show all the samples, the values in y-axis are randomly generated to reduce the overlap between samples with similar TACSTD2 expression levels.

**Supplementary Figure S7.** Flow cytometry analysis of TACSTD2 expression in MCF7 and MDA-MB-231, and the cells treated with 10µM forskolin and 100µM IBMX (FI) for 1 day (n=3). Black curve: untreated cells. Red curve: FI treated cells. Many more cells in the MDA-MB-231 than the MCF-7 cell line have TACSTD2 in the ON state.
